# Decreased expression of cytochrome P450 protein in non-malignant colonic tissue of patients with colonic adenoma

**DOI:** 10.1186/1471-230X-5-34

**Published:** 2005-11-10

**Authors:** Ina Bergheim, Christiane Bode, Alexandr Parlesak

**Affiliations:** 1 Hohenheim University (140), Department Physiology of Nutrition, Stuttgart, Germany; 2 Department of Pharmacology and Toxicology, University of Louisville Health Sciences Center, Louisville, KY, USA

## Abstract

**Background:**

Cytochrome P450 (CYP) enzymes in epithelial cells lining the alimentary tract play an important role in both the elimination and activation of (pro-)carcinogens. To estimate the role of cytochrome P450 in carcinogenesis of the colon, expression patterns and protein levels of four representative CYPs (CYP2C, CYP2E1, CYP3A4 and CYP3A5) were determined in colon mucosa of normal and adenomatous colonic tissue of patients with adenomas and disease-free controls.

**Methods:**

Expression of CYP2C, CYP2E1, CYP3A4, and CYP3A5 in colon mucosa of normal and adenomatous colonic tissue of patients with adenoma and disease-free controls was determined by RT-PCR. Protein concentration of CYPs was determined using Western blot.

**Results:**

With the exception of CYP3A5, expression of CYP mRNA was similar among groups and tissues (e.g. normal colon mucosa and adenoma). CYP3A5 mRNA expression was significantly higher in adenoma in comparison to normal tissue of patients with adenoma (~48%). When comparing protein concentrations of CYPs measured in adenomas with neighboring normal colonic mucosa no differences were found. However, in normal tissue of patients with adenomas, protein levels of CYP2C8, CYP3A4 and CYP3A5, but not that of CYP2E1, were significantly lower than in biopsies obtained from disease-free controls. Specifically, in normal colonic mucosa of patients protein concentrations of CYP2C8, CYP3A4, and CYP3A5 were ~86%, ~69%, and ~54%, respectively, lower than in disease-free controls.

**Conclusion:**

In conclusion, among other factors, the altered protein levels of certain CYPs (e.g. CYP2C8, CYP3A4 and CYP3A5) in colon mucosa might contribute to the development of neoplasia in the colon.

## Background

Cytochrome P450 enzymes (CYPs) play a key role in the oxidative, peroxidative and reductive metabolism of endogenous and exogenous compounds such as drugs, dietary components and alcohols (for review see [1].). However, CYPs not only function in the detoxification of xenobiotics but may also be involved in the activation of potential (pro-) carcinogens. Furthermore, it has been suggested that the local expression of CYPs in tumors is very important for the management of cancer since CYPs expressed in tumors may be involved in activation and/or inactivation of chemotherapeutic drugs (for review see [2].). The alimentary tract is exposed to a large variety of xenobiotics, including potential (pro-) carcinogens. Indeed, it has been proposed that extrahepatic tissue might play an important role in the CYP-mediated metabolism of xenobiotic compounds and therefore eventually the susceptibility of certain organs to the development of neoplasia (e.g. colon); however, knowledge on regulation and localization of CYP-mediated xenobiotic metabolism outside the liver is limited.

CYP is most abundant in liver; however, the main CYP families participating in the metabolism of xenobiotics (e.g. CYP1, 2, 3) are also expressed in extrahepatic tissues (for review see [3].). Kaminsky and Fasco [4]. have proposed that members of cytochrome P450 families 3 (e.g. CYP3A) and 2 (e.g. CYP2C), which are present in the small intestinal epithelium at high concentrations [5], protect the small intestine from carcinogenesis. Furthermore, it has been suggested that the absence of some of these microsomal enzymes in the colon may be involved in the comparably high incidence of carcinoma in this organ [6]. However, the availability of data on human colonic cytochrome P450 protein expression in unaffected as well as in adenomatous tissue of the colon is limited. Furthermore, some of the published findings are contradictory. For example, expression of mRNA for CYP3A4 and CYP3A5 has been found in human colorectal epithelium [7]. and in intestinal cell lines [8,9]. CYP2E1 protein has been detected in colon carcinoma cell lines and in the colon [10,11]. but not in human peritumoral or tumoral tissue [12]. Expression of CYP2C7, corresponding to human CYP2C8, and CYP2C (including CYP2C8-CYP2C19) in human and rat colon tissues has been reported [11,13,14].

CYPs might play a critical role not only in the development but also in treatment of colonic neoplasia. However, knowledge on CYPs in colon is limited. Therefore, the aim of the present study was to determine expression and protein concentrations of four representative CYPs (e.g. CYP2C, CYP2E1, CYP3A4, and CYP3A5) in macroscopically normal colonic tissue of subjects with and without colonic adenomas. As the expression of different xenobiotic-metabolizing enzymes may be altered throughout the development of neoplasia, which is generally accepted to follow the adenoma-carcinoma sequence, expression of these cytochrome P450 genes was also measured in colonic adenoma and compared to that of unaffected neighboring colonic tissue of the same subjects.

## Methods

### Subjects

The study protocol was approved by the Ethics Committee of the medical association Stuttgart, Germany, and followed the ethical guidelines of the 1975 Declaration of Helsinki. Informed consent was obtained from all subjects. Colonic adenoma and surrounding normal colon mucosa biopsies (each 5–10 mg) were obtained during routinely performed coloscopies from 25 patients, aged 50 – 70 years (Table 1). In addition, biopsy specimens of normal colon mucosa were obtained from 27 disease-free controls during routine endoscopy (age 50 – 70 years) displaying no signs of colonic neoplasia or other diseases of the colon. Immediately after removal of biopsies, colon mucosa specimens were frozen in liquid nitrogen and stored at -80°C. All patients completed a questionnaire concerning factors that may influence the expression of cytochrome P450 such as medication, smoking, and alcohol consumption (Table 2). Neither anthropometrics nor medication differed significantly between the patient group and that of disease-free controls. Patients and disease-free controls did not consume drugs known to interfere with/ or to induce cytochrome P450 enzymes investigated in this study.

### Tissues and isolation of total RNA and protein

All colon mucosa specimens were immediately frozen in liquid nitrogen after excision and stored at -80°C. Both total RNA and protein were isolated using Trizol reagent (Invitrogene, Gaithersburg, MD, USA).

### Immunoblot analysis

Antibodies used for the detection of CYP2E1 and CYP3A4 were a generous gift of Dr. M. Ingelman-Sundberg, Karolinska Institute, Stockholm, Sweden. Primary antibodies for the measurements of CYP2C8 and CYP3A5 were purchased from Chemicon, Inc. (Frankfurt, Germany). Microsomes and Supersomes were from cell lines/clones over-expressing human CYP2C8, CYP2E1, CYP3A4, and CYP3A5, respectively. They were used as internal standards for quantification and to test for cross reactivity of antibodies (Gentest Corporation, Woburn, MA, USA). Protein concentration was determined by the method of Bradford [15], using a commercial preparation (BioRad, Munich, Germany).

Twenty to 30 μg of total protein were separated by SDS-polyacrylamid gel electrophoresis and transferred to nitrocellulose membranes. To enable quantification of CYP protein, a serial dilution of the appropriate standard was proceeded identically. To ensure equal loading of samples, membranes were pre-stained with Ponceau red before blocking and incubation with antibodies. Membranes were blocked in 5% non-fat milk in Tris-buffered saline-Tween 20 (TBST, 0.01% v/v Tween 20) and probed with dilutions of primary antibodies in TBS, followed by an incubation with the secondary antibody. The protein/antibody complex was visualized by enhanced chemiluminescence (SuperSignal^® ^West Dura, Pierce, KTM, Bad Godesberg, Germany). Blots were evaluated (Camera LAS 1000, Fuji, USA) and densitometric analysis was performed using the software AIDA (Raytest, Isotopenmessgeraete, Straubenhardt, Germany). Signal intensities of the samples were adjusted to the intensities of the serially diluted standards. To ensure equal loading some blots were probed for β-actin (Sigma, St Louis, USA).

### Reverse transcription and PCR

The integrity and concentration of RNA was analyzed in a 1.2% agarose gel. First-strand complementary DNA was synthesized from 200 ng of total RNA using a First-Strand cDNA Synthesis Kit (Invitrogen, Gaithersburg, MD, USA). Sequences of primers are summarized Table 3. The PCR reaction consisted of 0.6 μl of cDNA, 10 x PCR buffer, 200 μM dNTPs (Boehringer, Mannheim, Germany), BSA (0.25 mg/ml), DMSO (2% v/v), 0.5 μM of specific primer and 0.5 U Taq-polymerase (Promega, Madison, WI, USA), and water to a final volume of 10 μl. For amplifications of the four cytochrome P450 cDNAs, PCR-conditions were as follows: 3 s at 94°C, 3 s at 45°C, 30 s at 72°C, for 32 cycles. Amplification of histone 3.3 was performed applying the following conditions: 3 s at 94°C, 3 s at 45°C, and 30 s at 72°C for 30 cycles. All PCR amplifications were carried out in triplicate in a Rapid Cycler (Idaho Tec., USA) within the linear range of the reaction. PCR products were separated in a 1.5% agarose gel, stained with ethidium bromide and photographed using a digital camera from Biometra (Goettingen, Germany). To ensure the success of PCR, human liver cDNA was used as a positive control.

### Statistical analysis

Results are presented as means ± standard error of the mean (SEM) unless otherwise indicated. Fisher's exact test was used to compare lifestyle data. The Mann-Whitney *U*-test was used for the comparison of relative mRNA concentration and protein levels measured in normal colon mucosa obtained from patients with adenoma and disease-free controls. Wilcoxon's *t*-test was used for the comparison of relative mRNA expression and protein concentration measured in normal colon mucosa and colonic adenoma of the same subjects. Differences accepted as significant had a significance level (*P*) of less than 0.05.

## Results

### Expression of CYP2C, CYP2E1, CYP3A4, and CYP3A5 mRNA in colon of patients with colonic adenoma and disease-free controls

**Figure 1:**
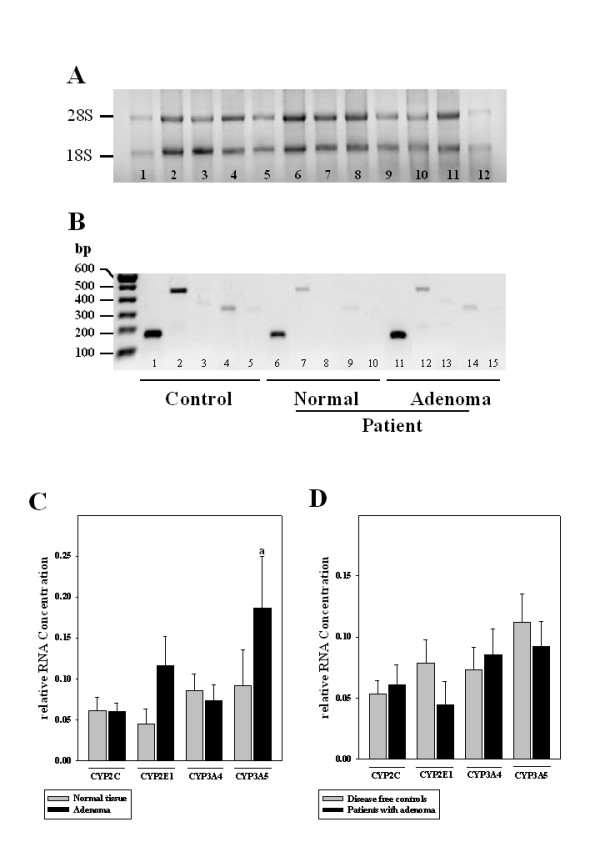
**CYP3A5, CYP2E1, CYP3A4, and CYP2C mRNA expression in colon mucosa of patients with adenoma and disease-free controls**. (A) Representative agarose gel of mRNA integrity. Lane 1 and 12 = positive control, lane 2–11 = RNA extracted from colonic mucosa biopsies. (B) Representative photomicrograph of RT-PCR products of two subjects. All measurements were carried out in triplicate. Lanes 1, 6, 11: Histone 3.3; Lanes 2, 7, 12: CYP3A5; Lanes 3, 8, 13: CYP2E1; Lanes 4, 9, 14: CYP3A4; Lanes 5, 10, 15 = CYP2C, bp = base pairs. (C) Quantitative analysis of CYP2C, CYP2E1, CYP3A4, and CYP3A5 mRNA expression in normal colon mucosa and in colonic adenoma of patients. Results are normalized to histone 3.3 expression. Data are means ± SEM, ^a^p < 0.05 compared to normal tissue. (D) Quantitative analysis of CYP2C, CYP2E1, CYP3A4, and CYP3A5 mRNA expression in normal colon mucosa of patients with colonic adenoma and disease-free controls. Results are normalized to histone 3.3 expression. Data are means ± SEM, ^a^p < 0.05 compared to disease-free controls.

High quality, non-degraded mRNA was obtained from normal tissue of 20 disease-free controls and unaffected tissue as well as adenomas of 18 patients with colonic adenoma. Expression of histone 3.3, which was used as housekeeping gene, was detected in all samples. Figure 1 depicts representative agarose gels of RNA integrity and results of RT-PCR measurements. Results of CYP2C, CYP2E1, CYP3A4, and CYP3A5 mRNA expression are summarized in Figures 1C and 1D. When comparing the relative mRNA concentration of CYP2C, CP2E1, CYP3A4, and CYP3A5 between adenomatous tissue and normal tissue of patients with adenoma, significant differences were only found for the expression of CYP3A5. Specifically, CYP3A5 mRNA expression was significantly higher by ~48% in adenomatous tissue in comparison to normal colonic tissue. No differences were found when comparing CYP expression in normal tissue of patients with adenoma and disease-free controls.

### Protein levels of CYP2C8, CYP2E1, CYP3A4, and CP3A5 in colon of patients with colonic adenoma and disease-free controls

**Figure 2:**
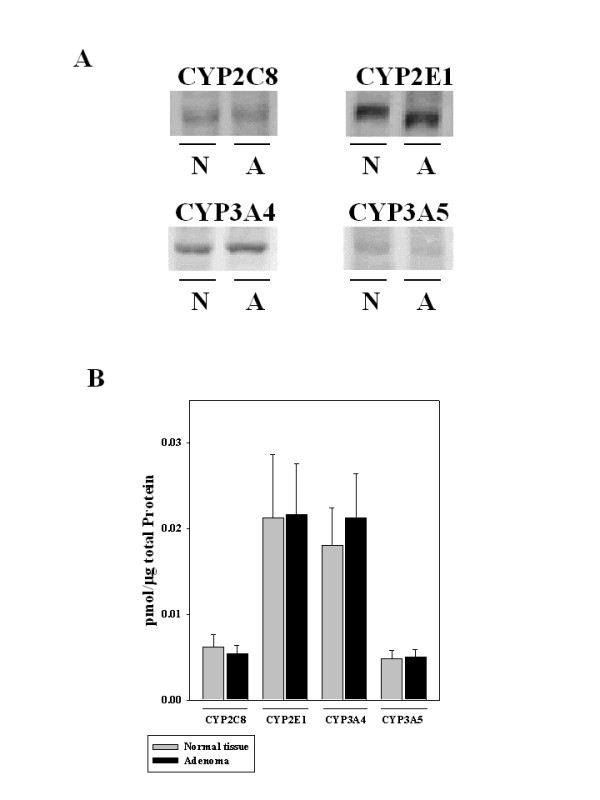
**Protein levels of CYP2C8, CYP2E1, CYP3A4, and CYP3A5 in macroscopically normal tissue and in adenoma of patients with colonic adenoma**. (A) Representative Western blots of CYP2C8, CYP2E1, CYP3A4, and CYP3A5 in macroscopically normal colon mucosa (= N) and adenoma (= A) of patients with colonic adenomas and (B) quantitative analysis of blots are shown. CYP2E1, CYP3A4, and CYP3A5 protein levels were determined in 25 patients with adenoma. CYP2C8 protein levels were determined in 19 cases. Data are means ± SEM

Western blot analyses of CYP2E1, CYP3A4, and CYP3A5 were performed with specimens obtained from 25 patients with colonic adenoma and 27 disease-free controls. Since higher protein concentrations were needed for the detection of CYP2C8, protein concentration of CYP2C8 was only determined in biopsies obtained form 19 cases and 12 disease-free controls. No differences were found when comparing mean protein levels of CYP2C8, CYP2E1, CYP3A4, and CYP3A5 measured in normal and neoplastic tissue of patients with adenoma. Representative Western blots and quantitative analysis of blots are depicted in Figure 2.

**Figure 3:**
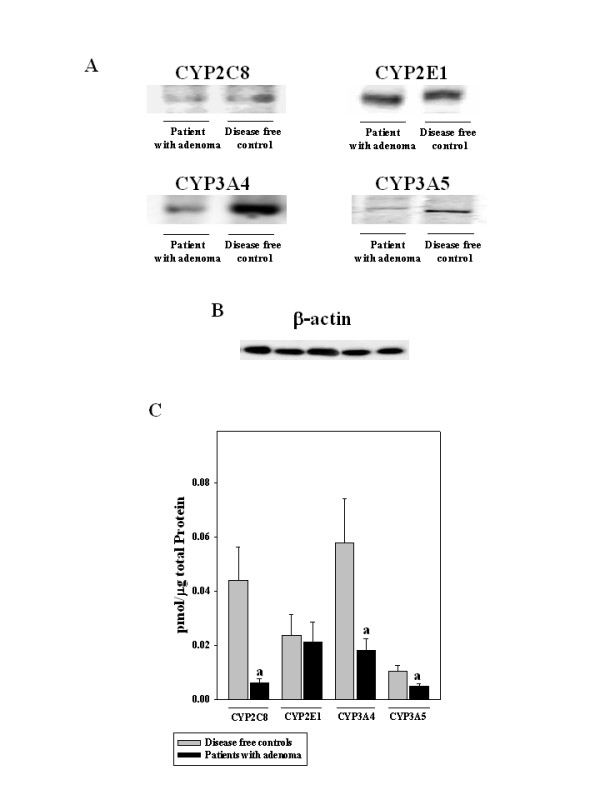
**Diminished protein levels of CYP2C8, CYP3A4, and CYP3A5 in normal colon mucosa of patients with colonic adenoma**. (A) Representative Western blots of CYP2C8, CYP2E1, CYP3A4, and CYP3A5 in normal colon mucosa from patients with colonic adenomas and disease-free controls. (B) Representative Western blot of β-actin protein in normal mucosa of patients with colonic adenoma and disease-free controls. (C) Quantitative analysis of blots. CYP2E1, CYP3A4, and CYP3A5 protein levels were determined in 25 patients with adenoma und 27 disease-free controls. CYP2C8 protein levels were determined in 19 cases and 12 disease-free controls. Data are means ± SEM, ^a^p < 0.05 compared to disease-free controls.

In addition, protein levels of CYPs were also determined in colon mucosa biopsies of disease-free controls and compared with those determined in macroscopically normal tissue of patients with colonic adenoma. Figure 3 depicts representative Western blots and quantitative analysis of protein.

Protein levels of CYP2E1 in mucosal biopsies obtained from normal colon mucosa of patients with adenoma did not differ significantly from those measured in disease-free controls. In contrast, mean protein level of CYP2C8 was significantly lower in unaffected, macroscopically normal colon mucosa obtained from patients with adenoma than in samples obtained from disease-free controls. Specifically, protein concentration of CYP2C8 in colon mucosa obtained from patients with adenoma was ~85% lower than in tissue obtained from disease-free controls. Similar results were found when comparing protein levels of CYP3A4 and CYP3A5 between patients with adenoma and disease-free controls. CYP3A4 protein concentration was ~69% lower in colon mucosa of patients with adenoma in comparison to disease-free controls. Protein level of CYP3A5 was ~54% lower in biopsies obtained from patients with adenoma when compared to disease-free controls.

### Relation of protein levels and mRNA expression pattern of CYPs in normal colonic mucosa of patients with adenoma and disease-free controls

**Figure 4:**
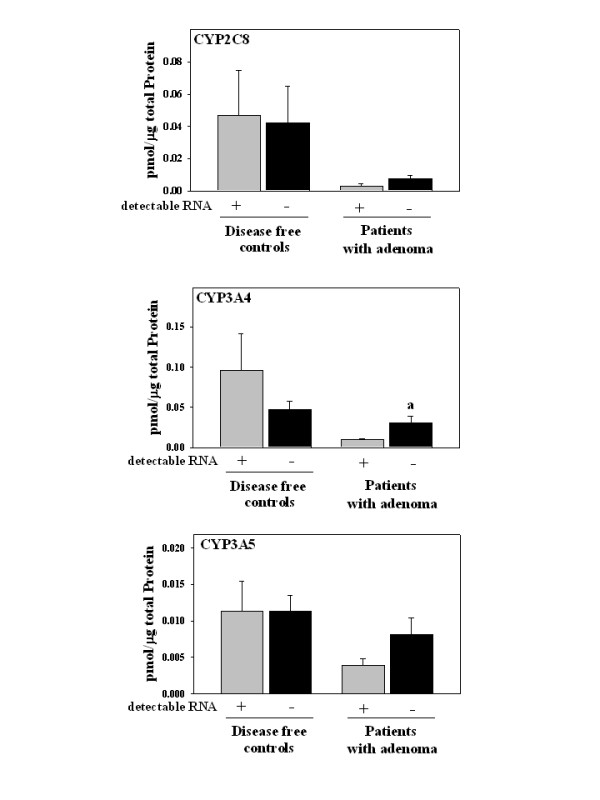
**Relation of CYP2C8, CYP3A4, CYP3A5 protein levels and mRNA expression pattern in normal colon mucosa of cases and disease-free controls**. Comparison of protein levels of CYP2C8, CYP3A4, and CYP3A5 of patients and disease-free controls, respectively, with detectable mRNA expression and those with no detectable mRNA expression of the respective CYP. Data are mean ± SEM. ^a^p < 0.05 compared to disease-free controls.

To further investigate whether differences found in protein levels of CYPs between normal tissue of patients with adenoma and disease-free controls are related to the mRNA expression of CYPs, protein levels of CYP2C8, CYP3A4, and CYP3A5 of subjects with detectable mRNA expression were compared to those with undetectable mRNA expression of the respective CYP. CYP2E1 was excluded from this comparison, since CYP2E1 expression was only detected in three disease-free controls and two patients with adenoma. No comparison was performed with adenomatous tissue since the sample sizes were too small (n < 5) for most of the CYPs investigated. Results are summarized in Figure 4.

Despite the fact that expression of CYP2C was detected in ~50 % of patients with adenoma and disease-free controls, no differences were found when comparing protein levels of patients with adenoma and disease-free controls with detectable and undetectable mRNA expression. Similar, protein levels of CYP3A4 in disease-free controls with undetectable mRNA expression did not differ from those with detectable mRNA expression of CYP3A4. In contrast, CYP3A4 protein levels of patients with adenoma with undetectable mRNA expression of CYP3A4 were significantly higher when compared to those with detectable mRNA expression. Specifically, protein levels were ~68 % higher in patients with adenoma with undetectable mRNA expression. In disease-free controls, protein concentration of CYP3A5 did not differ between disease-free controls with detectable und undetectable CP3A5 mRNA expression. Similar to CYP3A4, protein levels of CYP3A5 were ~46 % higher in patients with undetectable mRNA expression in comparison to those with detectable mRNA expression of CP3A5. However, due to a large inter-individual variability differences were not statistically significant (p = 0.098).

## Discussion

### Protein levels of CYPs are decreased in normal tissue of patients with colonic adenoma

Carcinogens and pro-carcinogens present in the diet are critical environmental factors influencing the development of carcinoma in the large intestine. Cytochrome P450-mediated metabolism of (pro-)carcinogens has been shown – depending upon the compound – to either result in detoxification or toxification (for review see [1]) and might therefore have potential impact on the development of neoplasia. Furthermore, most human CYPs have been found to be genetically polymorphic and these polymorphisms may affect the enzyme expression and activity subsequently leading to an increased risk to develop several forms of cancer but also effecting treatment (for review see [16]). Only a few extensive studies on the mRNA and protein expression of cytochrome P450 in colonic tissue and colonic adenoma have been performed thus far. The presence of CYP2C-, CYP2E- and CYP3A- in normal and in neoplastic colonic mucosa along with substantial inter-individual variability has been reported by others before [12,14,17,18]. However, some of the available data are contradictory and most studies either determined mRNA expression or protein levels [14,18]. For example, Yokose et al. [14] showed the presence of CYP2C (CYP2C8-CYP2C19) protein in both unaffected and neoplastic human colon mucosa using immunohistochemical methods. In contrast, Western blot analyses of de Waziers et al. [18] and Massaad et al. [12], who both used conventional immunoperoxidase staining procedures, showed neither cytochrome P450 2C8-10 nor 2E1 to be present in normal colon mucosa and in colon carcinoma. McKay et al. [17], who used immunohistochemical methods, detected CYP3A protein more frequently in neoplastic tissue in colon mucosa specimens of patients with neoplasia than in the morphologically normal mucosa of these patients. Expression of CYP3A4 and CYP3A5 has been reported for human colon mucosa [19,20]. In the present study, the expression and protein levels of four representative CYPs were determined in macroscopically normal colon mucosa and adenoma of patients with adenoma and disease-free controls. At the level of mRNA expression, only the expression of CYP3A5 was found to be significantly altered between neoplastic tissue and normal, unaffected mucosa of patients with adenoma. However, as reported by others before, mRNA expression of all four CYPs varied extensively between individuals even though the expression of the housekeeping gene histone 3.3 was detected in all samples and was used for normalization. At the level of protein, CYP2C8, CYP3A4, and CYP3A5 protein concentration was found to be significantly lower in normal tissue of patients with adenoma than in colon mucosa of disease-free controls. Even though previous studies have indicated that the abundance of CYP protein is altered between neoplasia and normal tissue [17], in the present study protein levels of CYPs were found to be similar in neoplasia and normal tissue of patients with adenoma. Taken together, these data suggest that CYP expression not only varies extensively between individuals but that protein levels of some CYPs (e.g. CP2C8, CYP3A4, and CYP3A5) are considerably lower in normal tissue of patients with adenoma in comparison to those of disease-free controls.

### CYP protein levels and mRNA expression are not related in normal colonic tissue

It has been suggested that expression of CYPs is not solely regulated at the level of gene transcription [11]. This is supported by the results of animals studies, reporting a dissociation of mRNA expression and protein levels of CYP2C7 (corresponding to human CYP2C8) and CYP2E1 in rat colon mucosa as well as CYP3A4 and CYP3A5 in duodenum and kidney. Furthermore, *in vitro *studies performed in rat hepatocytes indicate that CYP2E1 is regulated by posttranscriptional ligand-dependent stabilization of the enzyme [21]. Similar mechanisms have been described for CYP3A in rats and humans [22,23]. Using cultured hepatocytes it also has been showed that only ~60–70% of mRNA encoding for CYP2E1 is translated [24]. Indeed, in the present study, no relation with respect to protein levels of subjects with detectable and undetectable mRNA expression of the CYPs was found. Instead, protein levels of CYP2C8, CYP3A4, and CYP3A5 did not differ in disease-free controls. In contrast, in normal tissue of patients with adenomas protein levels of CYP3A4 and CYP3A5 were contrary to mRNA expression pattern. Taken together, these data suggest that in colon mucosa the expression of CYP2C8, CYP3A4, and CYP3A5 is not solely regulated at the level of transcription and that the mechanism of regulation, at least for some CYPs (e.g. CYP3A4 and CYP3A5), might differ between patients with colonic adenoma and healthy subjects.

## Conclusion

In summary, inter-individual variability along with a substantial dissociation of mRNA expression pattern and protein levels seems to be a characteristic of CYP expression in the colon as has been reported in part by others before [12,14,17,18]. However, in the present study protein levels of CYP2C8, CYP3A4, and CYP3A5 were found to be significantly lower in normal unaffected colonic mucosa of patients with colonic adenoma in comparison with disease-free controls. Although the metabolic implications of these differences remain to be determined, reduced levels of some CYPs might result in an altered metabolism of xenobiotics and therefore contribute to the development of neoplasia in the large intestine. Future studies will address this possibility.

## Competing interests

The author(s) declare that they have no competing interests.

## Authors' contributions

IB has made substantial contributions to acquisition of data, the biochemical analysis, and the drafting of article. CB has made substantial contribution to conception and design as well as the interpretation of data. AP has been involved in the design, the drafting of the article, and revised it critically for intellectual content. All authors have given final approval of the version to be published.

## Pre-publication history

The pre-publication history for this paper can be accessed here:



## Figures and Tables

**Table 1:** Origin of tissue specimen and histology of colonic adenomatous tissue samples.

	**Patients with adenoma**		**Disease-free controls**
	**Normal tissue**	**Adenoma**	**Normal tissue**
**Ascending colon**	8	7	8
**Descending colon**	8	12	5
**Sigmoid colon and rectum**	9	6	14

**Tubular adenoma**	-	12	-
**Tubular villious adenoma**	-	10	-
**Villious adenoma**	-	3	-

**Table 2:** Clinical data of patients with adenoma and disease-free controls

	**Patients with adenoma**	**Disease-free controls**
Number (female/male)	25 (9/16)	27 (12/15)
Age	61 ± 5	59 ± 6
Body-mass index (BMI)	26.9 ± 4.8	26.4 ± 4.4
Cigarette usage: yes/no (number/d)	8/17 (2.9 ± 6.9)	5/22 (2.1 ± 6.5)
Alcohol consumers (more than 10 g/d): yes/no	8/17	4/23
Average alcohol intake (g/d) of consumers	15.1 ± 16.5	10.5 ± 13.5
Medication (yes/none)	15/10	17/10

All data are expressed as means ± SD.

**Table 3:** Primers used for RT-PCR analysis

	**Sense primer**	**Antisense primer**	**PCR product (bp)**	**Reference**
**Histone 3.3**	GCGTGCTAGCTGGATGTCTT	CCACTGAACTTCTGATTCGC	150	[25].
**CYP2C8-19**	GCTAAAGTCCAGGAAGAGATTGA	TCCTGCTGAGAAAGGCATGAAGT	332	[26].
**CYP2E1**	AGCACAACTCTGAGATATGG	ATAGTCACTGTACTTGAACT	365	[26].
**CYP3A4**	CCAAGCTATGCTCTTCACCG	TCAGGCTCCACTTACGGTGC	324	[27].
**CYP3A5**	TGTCCAGCAGAAACTGCAAA	TTGAAGAAGTCCTTGCGTGTC	472	[27].
